# Higher central circadian temperature amplitude is associated with greater metabolite rhythmicity in humans

**DOI:** 10.1038/s41598-024-67297-y

**Published:** 2024-07-22

**Authors:** Daniel P. Windred, Clare Anderson, Katherine J. Jeppe, Suzanne Ftouni, Leilah K. Grant, Brunda Nijagal, Shantha M. W. Rajaratnam, Malcolm McConville, Dedreia Tull, Steven W. Lockley, Sean W. Cain, Andrew J. K. Phillips

**Affiliations:** 1 Flinders Health and Medical Research Institute (Sleep Health), Flinders University, Bedford Park, SA Australia; 2 School of Psychological Sciences, Monash University, Clayton, VIC Australia; 3 School of Psychology, Centre for Human Brain Health, University of Birmingham, Edgbaston, UK; 4 Monash Proteomics and Metabolomics Platform, Department of Medicine, School of Clinical Sciences, Monash University, Clayton, VIC Australia; 5 Division of Sleep and Circadian Disorders, Departments of Medicine and Neurology, Brigham and Women’s Hospital, Boston, MA USA; 6 Division of Sleep Medicine, Harvard Medical School, Boston, MA USA; 7 Metabolomics Australia, Bio21 Molecular Science and Biotechnology Institute, University of Melbourne, Parkville, VIC Australia

**Keywords:** Biological rhythms, Peripheral oscillators, Circadian organization, Circadian clock, Core body temperature, Medical research, Predictive markers, Metabolomics, Neurophysiology

## Abstract

Robust circadian rhythms are essential for optimal health. The central circadian clock controls temperature rhythms, which are known to organize the timing of peripheral circadian rhythms in rodents. In humans, however, it is unknown whether temperature rhythms relate to the organization of circadian rhythms throughout the body. We assessed core body temperature amplitude and the rhythmicity of 929 blood plasma metabolites across a 40-h constant routine protocol, controlling for behavioral and environmental factors that mask endogenous temperature rhythms, in 23 healthy individuals (mean [± SD] age = 25.4 ± 5.7 years, 5 women). Valid core body temperature data were available in 17/23 (mean [± SD] age = 25.6 ± 6.3 years, 1 woman). Individuals with higher core body temperature amplitude had a greater number of metabolites exhibiting circadian rhythms (R^2^ = 0.37, p = .009). Higher core body temperature amplitude was also associated with less variability in the free-fitted periods of metabolite rhythms within an individual (R^2^ = 0.47, p = .002). These findings indicate that a more robust central circadian clock is associated with greater organization of circadian metabolite rhythms in humans. Metabolite rhythms may therefore provide a window into the strength of the central circadian clock.

## Introduction

The circadian system plays a fundamental role in human health. Circadian disruption is associated with a wide range of poor health outcomes^1–3^. The circadian system is hierarchically structured, with a central circadian clock in the suprachiasmatic nucleus (SCN)^4^ that orchestrates the rhythms of peripheral (non-SCN) circadian clocks throughout the brain and body^5^. The SCN tightly controls endogenous circadian rhythmicity in core body temperature, with highest temperature in the late day or early evening and lowest temperature in the late night or early morning^6^. Studies in animals have demonstrated that temperature cycles organize the timing of peripheral clocks, which are temperature sensitive^5,7–9^. It is not known whether temperature cycles play a similar role in organizing other circadian rhythms in humans.

Peripheral circadian rhythms have been studied using a wide array of methodologies, including metabolomic and transcriptomic approaches^5^. In humans, metabolomic studies have demonstrated circadian rhythmicity in many classes of metabolites, including amino acids^10^, acylcarnitines^11^, lipids^12^, and highly polar metabolites^13^. Alterations in circadian metabolite rhythms, and other peripheral rhythms, may reflect disruption of the central circadian clock. For example, metabolomic, transcriptomic, and proteomic rhythms can be altered by experimental exposure to circadian misalignment^14–17^, which can also disrupt central circadian rhythms. However, further research is required to clarify relationships between central and peripheral rhythms, particularly due to potential masking of circadian rhythms when participants are not studied under controlled conditions.

Core body temperature measured under constant routine conditions is considered a gold-standard measure of the state of the central circadian clock in human research^18–20^. Variations in core body temperature amplitude have been assessed under a variety of experimental and observational paradigms, but their relationship with other rhythms throughout the body is not well understood. Preliminary evidence from a small sample (n = 6) suggests that core body temperature amplitude relates to the observed number of peripheral rhythms. However, participants were not studied under constant routine conditions, meaning the endogenous central and peripheral rhythms would both have been subject to masking.

We measured core body temperature amplitude using a constant routine protocol that controlled for masking effects of sleep, movement, and environmental factors on core body temperature. We then examined the relationship between core body temperature amplitude and rhythms of 929 polar metabolites measured from blood plasma.

## Results

Blood plasma metabolite rhythms and core body temperature were collected in 23 participants (age [M ± SD] = 25.4 ± 5.7 years, 5 women in the follicular phase of the menstrual cycle and 18 men). Blood plasma samples were collected every 2 h during a 40-h constant routine protocol. A total of 929 individual metabolites were isolated and detected by high performance liquid chromatography-mass spectrometry (HPLC–MS) in each plasma sample, providing 386,464 metabolite data points for analysis (see “Experimental protocol”). Metabolites were identified as having a circadian rhythm by assessing the fit of a cosine function with 24-h period for each metabolite within each individual**.** A total of 46 of 929 metabolites were identified as circadian metabolites, defined as those exhibiting a significant fit in > 65% of participants (15/23).

**Figure 1:**
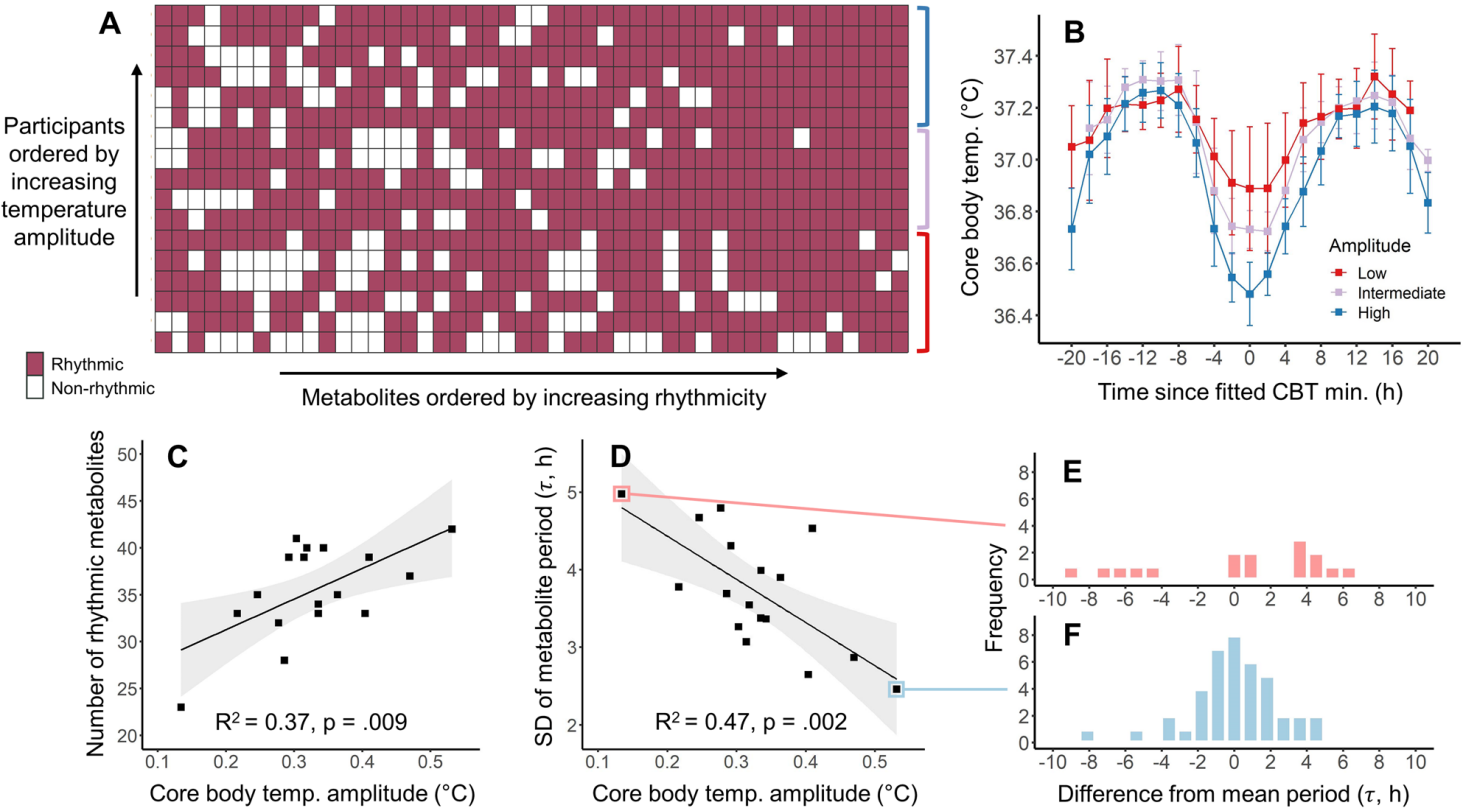
(**A**) Circadian metabolites that were significantly rhythmic for each participant. Rows represent the 17 participants, ordered by core body temperature amplitude, and columns represent the 46 circadian metabolites, ordered by number of significantly rhythmic instances across participants. (**B**) Core body temperature, split by tertiles into low (red; N = 6), moderate (purple; N = 5), and high (blue; N = 6) circadian amplitude. Color groupings map to rows indicated by the colored brackets in panel A. Points and error bars represent mean and standard deviation of core body temperature within 2-h bins relative to core body temperature minima. (**C**) Higher core body temperature amplitude was associated with a greater number of rhythmic circadian metabolites. (**D**) Higher core body temperature amplitude was associated with lower within-individual variability in metabolite periods. Linear models (black line) and corresponding 95% confidence intervals (gray shading) are shown for both associations. Variability in the metabolite period distribution is shown for the participants with the lowest (**E**) and highest (**F**) core body temperature amplitudes, plotted as the difference in period for each rhythmic metabolite from each participant’s mean metabolite period.

 Core body temperature was measured using ingestible telemetric pills (BodyCAP, Equivital, Cambridge, UK) over the 40-h constant routine, with amplitude estimated by a two-harmonic fit. Six participants were excluded due to low-quality or missing core body temperature data, leaving 17 participants (age [M ± SD] = 25.6 ± 6.3 years, 1 woman) in final analyses (see “Methods”). Average core body temperature amplitude was 0.33 ± 0.09 °C (M ± SD), with a range of 0.13–0.53 °C. Differences in core body temperature amplitude between participants were primarily driven by differences in the depth of the core body temperature minimum (see Fig. 1B). Higher core body temperature amplitude was associated with lower fitted core body temperature minimum (R^2^ = 0.80, F(1,15) = 59.64, p < 0.0001), whereas amplitude was not associated with fitted core body temperature maximum (R^2^ = 0.003, F(1,15) = 0.05, p = 0.83).

### Higher core body temperature amplitude was associated with greater prevalence of metabolites with circadian rhythmicity

The number of circadian metabolites that were identified as being significantly rhythmic averaged 35.5 ± 5.0 (M ± SD; 3.8% of all metabolites) across the 17 participants, and ranged from 23 to 42. Higher core body temperature amplitude was associated with a greater number of circadian metabolites that were significantly rhythmic within each individual (R^2^ = 0.37, F(1,15) = 8.90, p = 0.009; see Fig. 1A–C). A 0.1 °C higher core body temperature amplitude predicted the presence of 3.3 additional rhythmic circadian metabolites. This result was robust to our definition of a circadian metabolite across a wide range of cut-offs (see “Sensitivity analyses”) and also robust to exclusion of the female participant (R^2^ = 0.38, p = 0.01). Lower core body temperature minimum was associated with greater number of rhythmic metabolites (R^2^ = 0.23, p = 0.048), but core body temperature maximum was not associated with the number of rhythmic metabolites.

### Higher core body temperature amplitude was associated with less variable periods of metabolite rhythms

In any hierarchical system of oscillators, where peripheral clocks are collectively entrained by a central clock^5^, lower amplitude of the central clock would be theoretically expected to cause more variable rhythms among the peripheral clocks due to weaker entrainment, or decoupling of some peripheral rhythms from the central clock. In either of these theoretical cases, lower central circadian amplitude would thus be expected to associate with greater variability in the periods of peripheral rhythms within the same individual. We therefore tested the hypothesis that lower core body temperature amplitude is associated with greater variability of metabolite rhythm periods. Metabolite signals were fit by a cosine function with period as a free parameter. Across participants, an average of 34.2 ± 6.6 metabolites were significantly rhythmic (range: 16–42; 3.7% of all metabolites), with free-fitted periods from 16 to 32 h and average period of 24.3 ± 1.1 h (M ± SD). Metabolite period variability was defined as the standard deviation of free-fitted periods across all rhythmic metabolites in an individual, and averaged 3.72 ± 0.8 h (M ± SD). We found that lower core body temperature amplitude was associated with greater metabolite period variability (R^2^ = 0.47, F(1,15) = 13.39, p = 0.002, see Fig. 1D–F). A 0.1 °C lower core body temperature amplitude predicted 0.47 h higher metabolite period variability. This result was robust to our definition of a circadian metabolite across a wide range of cut-offs (see “Sensitivity analyses”) and also robust to exclusion of the female participant (R^2^ = 0.47, p = 0.003). Lower core body temperature minimum was associated with lower metabolite period variability (R^2^ = 0.37, p = 0.01), but core body temperature maximum was not associated with metabolite period variability. We note that greater variability in free-fitted periods could be reflective of peripheral oscillators with a greater range of intrinsic periods, weaker entrainment, and/or more stochastic waveforms leading to less certain period estimates.

### Sensitivity analyses

To test the robustness of our findings, the relationship between core body temperature amplitude and the number of rhythmic circadian metabolites was re-assessed, changing our definition of a circadian metabolite. We varied the cut-off for the percentage of participants exhibiting significant rhythms for each metabolite between 4.3% (1/23 participants) and 100%. We found that there were significant, robust relationships (R^2^ ≥ 0.25, p < 0.05) between core body temperature amplitude and the number of rhythmic metabolites for metabolite cut-offs between 52% (12/23) and 96% (22/23) of participants (Table 1). Similarly, we tested the sensitivity of the relationship between core body temperature amplitude and intra-individual variability in free-fitted circadian metabolite period to our definition of a circadian metabolite. Cut-offs in the range from 43% (10/23) to 83% (19/23) of participants resulted in robust and statistically significant relationships (Table 1). Relationships of core body temperature amplitude with the number of rhythmic metabolites and metabolite period variability were non-significant but exhibited positive and negative trends, respectively, for cut-offs below 52% (12/23) and 43% (10/23).

**Table 1:** Relationships of core body temperature amplitude with metabolite rhythmicity across potential definitions of ‘circadian’ metabolites.

Rhythmicity cut-off^a^	Model outcome: Number of rhythmic metabolites				Model outcome: Intra-individual metabolite period variability				Metabolites classified as rhythmic due to type 1 error (%)^b^
	Estimate (SE)	F (1,15)	p-value	R^2^	Estimate (SE)	F (1,15)	p-value	R^2^	
≥ 1/23	89.0 (393)	0.0514	0.824	0.0034	− 2.67 (2.75)	0.948	0.346	0.059	69.23
≥ 2/23	91.5 (386)	0.0561	0.816	0.0037	− 2.67 (2.74)	0.946	0.346	0.059	32.04
≥ 3/23	101 (358)	0.0789	0.783	0.0052	− 2.72 (2.72)	0.997	0.334	0.062	10.52
≥ 4/23	101 (286)	0.125	0.729	0.0082	− 2.80 (2.72)	1.07	0.318	0.066	2.60
≥ 5/23	84.0 (204)	0.169	0.687	0.011	− 2.85 (2.66)	1.14	0.302	0.071	0.50
≥ 6/23	69.5 (122)	0.326	0.576	0.021	− 3.04 (2.52)	1.46	0.246	0.088	0.08
≥ 7/23	66.7 (63.8)	1.09	0.313	0.068	− 3.22 (2.47)	1.69	0.213	0.10	0.01
≥ 8/23	52.3 (37.0)	2.00	0.178	0.12	− 3.53 (2.32)	2.32	0.149	0.13	0.00
≥ 9/23	50.7 (26.7)	3.61	0.0769	0.19	− 4.27 (2.10)	4.13	0.0601	0.22	0.00
≥ 10/23	41.2 (22.1)	3.46	0.0825	0.19	− 4.54 (1.71)	7.05	0.0180	0.32	0.00
≥ 11/23	33.2 (20.4)	2.64	0.125	0.15	− 4.43 (1.41)	9.91	0.00664	0.40	0.00
≥ 12/23	35.7 (16.1)	4.92	0.0423	0.25	− 4.23 (1.45)	8.57	0.0104	0.36	0.00
≥ 13/23	30.1 (15.0)	4.05	0.0625	0.21	− 4.75 (1.49)	10.2	0.00610	0.40	0.00
≥ 14/23	33.7 (11.3)	8.83	0.00952	0.37	− 4.67 (1.45)	10.4	0.00571	0.41	0.00
≥ 15/23	32.7 (11.0)	8.90	0.00927	0.37	− 5.57 (1.52)	13.4	0.00233	0.47	0.00
≥ 16/23	30.0 (10.0)	8.89	0.00932	0.37	− 5.31 (1.65)	10.3	0.00579	0.41	0.00
≥ 17/23	19.4 (7.04)	7.60	0.0147	0.34	− 6.24 (2.15)	8.43	0.0109	0.36	0.00
≥ 18/23	16.4 (6.13)	7.18	0.0171	0.32	− 6.75 (2.40)	7.93	0.0130	0.35	0.00
≥ 19/23	13.2 (4.65)	8.02	0.0126	0.35	− 6.02 (2.32)	6.77	0.0201	0.31	0.00
≥ 20/23	9.38 (3.49)	7.24	0.0167	0.33	− 4.49 (2.48)	3.29	0.0896	0.18	0.00
≥ 21/23	7.02 (2.74)	6.56	0.0217	0.30	− 4.76 (2.53)	3.55	0.0791	0.19	0.00
≥ 22/23	4.54 (1.21)	14.2	0.00188	0.49	− 0.32 (2.82)	0.0129	0.911	0.00086	0.00

Data are estimates, statistical significance, and effect sizes of linear regression models predicting number of rhythmic metabolites, and intra-individual metabolite period variability, from core body temperature amplitude.

^a^Rhythmicity cut-offs were used to define sets of ‘circadian’ metabolites. Each metabolite was required to exhibit significant circadian rhythmicity in ≥ N/23 participants for inclusion as a ‘circadian’ metabolite.

^b^Expected type 1 errors were simulated across 1000 sets of 929 metabolites*23 participants, and average percentage of metabolites defined as ‘circadian’ due to expected type 1 error alone were calculated for each rhythmicity cut-off.

## Discussion

The core body temperature rhythm has been proposed as an entraining signal for peripheral circadian clocks in non-human mammals^5,8^. We found that higher core body temperature amplitude in humans was associated with a greater prevalence of circadian metabolite rhythms and with lower variability in the periodicity of these metabolite rhythms. These findings suggest that core body temperature rhythms may play an important role in organizing metabolite rhythms in humans.

To our knowledge, the relationship between human core body temperature amplitude and circadian metabolite rhythmicity has not been previously studied. Our findings are consistent with one study that reported an association between diurnal core body temperature amplitude and rhythmicity of peripheral transcripts in shift workers^21^. Our findings are also consistent with studies demonstrating altered or reduced peripheral rhythmicity in transcripts^14^, proteins^17^, and metabolites^15,16^ following experimentally induced circadian misalignment, which would be predicted to attenuate endogenous and/or diurnal core body temperature amplitude^18,22^. Disruption of circadian rhythms has been linked to a wide range of negative health outcomes in humans^1,23^. For example, modeled circadian amplitude has recently been shown to predict incidence of type 2 diabetes^2^. Based on our data, we speculate that health effects could be mediated by reduced core body temperature amplitude leading to disorganization of peripheral metabolite rhythms. 

Individual differences in core body temperature amplitude were found in this study to be primarily due to differences in the depth of the temperature minimum. Temperature maxima were relatively consistent across participants and did not contribute to the relationship between core body temperature amplitude and circadian metabolite rhythmicity. If temperature plays a causal role in organizing metabolite rhythms, this finding has important implications. While endogenous rhythms in core body temperature are under the control of the central circadian clock, diurnal temperature rhythms in daily life can be significantly augmented or dampened by non-circadian masking factors including food intake^24^, menstrual phase^25^, physical activity, and sleep^26^, thereby aiding or opposing central circadian control of metabolite rhythms. For example, nocturnal sleep lowers the core body temperature minimum below its endogenous circadian minimum, which we hypothesize would lead to more robust metabolite rhythms in people with regular, high-quality sleep that is aligned with the biological night, due to amplification of the endogenous core body temperature signal. These findings may also have important implications for circadian health in the context of aging, where core body temperature amplitude is reduced, sleep is significantly disrupted, and higher risk of cardiometabolic morbidity is observed^27^.

We found that relationships between core body temperature amplitude and metabolite rhythmicity were most robust for metabolites that were rhythmic across most participants. Metabolites with significant cosine fits in a minority of participants did not contribute to amplitude-rhythmicity relationships. This finding could be due to the higher likelihood of false-positive rhythmicity classification for metabolites with significant cosine fits in smaller numbers of individuals. Additionally, unmeasured individual-level factors may contribute to inter-individual heterogeneity in rhythmic metabolite species. For example, inter-individual differences in gut microbiota^28^, which are known to influence circadian rhythms in peripheral organs and metabolic pathways^29^, could plausibly contribute to heterogeneity in rhythmic metabolite expression across individuals. We note that substantial heterogeneity in metabolite rhythmicity across individuals has also been observed in previous work^12^. If the state of the central pacemaker is reflected in the human metabolome, this information may be captured within a subset of species that exhibit group-level rhythmicity.

This study had several limitations. First, relationships between metabolite rhythmicity and core body temperature amplitude were correlational. Experimental work will be required to determine whether metabolite rhythmicity changes after suppression of endogenous core body temperature amplitude, which can be achieved by exposure to bright light close to the core body temperature nadir^18,19^. Second, it is unclear whether relationships between core body temperature amplitude and metabolite rhythmicity will generalize to free-living conditions. Our constant routine protocol was designed to capture the endogenous circadian components of core body temperature and metabolite rhythms, but these rhythms may vary due to free-living patterns of nutritional intake, physical activity, and sleep. Third, we note that periodicity is generally estimated across multiple circadian cycles, whereas we estimated free-fitted metabolite period from approximately 1.5 circadian cycles. Despite this limitation, the average free-fitted metabolite period of 24.3 h closely matched the established intrinsic period of the human central circadian pacemaker^30^. Finally, participants were predominantly men. While women were assessed in their follicular phase, future work should also explore whether temperature-metabolite relationships remain robust with changing menstrual phase, which influences core body temperature nadir and amplitude^31^.

In humans, the state of the central circadian clock is described by two key parameters: phase and amplitude^18,19^. Accurate measures of phase are routinely used in human circadian research^32,33^, but comparable measures of amplitude are not, due to the difficulty in obtaining non-masked, endogenous cycles. Assessing phase but not amplitude provides an incomplete picture of the circadian system. Low-burden estimation of central circadian rhythms may be possible, as demonstrated in research estimating central circadian phase from multivariate data using a small number of samples^34–39^. Our results suggest that rhythms in peripheral markers, such as metabolites, could reflect the strength of the central circadian clock and may therefore provide a key measure for understanding circadian health.

## Methods

### Recruitment and screening

Participants were recruited according to the following criteria: (1) aged 20–45 years, (2) no medical, psychiatric, or sleep disorders, (3) did not travel across time zones within three months prior to laboratory admission, (4) did not work rotating shifts within the prior 3 years, (5) were fluent in English, and (6) did not use prescription or over-the-counter medications, nicotine, alcohol, or caffeine for three weeks prior to laboratory admission. Recent recreational drug use and pregnancy were screened via urine test at time of admission, and a breath test was administered to screen for alcohol use. Female participants commenced the laboratory protocol during the follicular phase of their menstrual cycle (self-reported)^31^.

Written informed consent was obtained prior to study enrolment via interview. Ethics approval was received from the Monash University Human Research Ethics Committee (CF14/2790—2014001546), and all procedures were conducted in accordance with the Declaration of Helsinki.

### Experimental protocol

Core body temperature and metabolite rhythms were assessed during a 40-h constant routine protocol to control for factors that mask endogenous rhythms. Participants remained awake, in a constant semi-recumbent position (~ 45° angle) under dim light (2.84 ± 0.72 lx) for the protocol duration. The laboratory environment was free of time cues, sound-attenuated, and temperature regulated (22.0 ± 0.8 °C). Participants were fed identical isocaloric snacks every hour, composed of ~ 20% protein, ~ 33% fat and ~ 46% carbohydrate. Regular self-selected sleep schedules (8:16 h) were maintained for two weeks at home prior to laboratory admission, confirmed by wrist-worn actigraphy, sleep diaries, and daily time-stamped phone calls. The study protocol commenced with two nights of sleep in the laboratory, timed according to each participant’s regular 8:16 h schedule, and lighting was maintained at 101.8 ± 38.5 lx during wake and 0 lx during sleep. Constant routine protocol commenced upon awakening on day three.

Ingestible telemetry pills (BodyCAP, Equivital, Cambridge, UK) had a temperature measurement range of 25–50 °C, logged data in 15 s epochs, and transmitted data to a torso-worn Sensor Electronics Module linked with Equivital LifeMonitor Software, for real-time temperature monitoring. Pills were administered prior to sleep on day two, approximately 8 h before constant routine commencement (N = 15), or at the commencement of constant routine on day three (N = 8). A second pill was administered in 5 of 23 participants, due to signal dropout coinciding with bowel movement. These differences between participants were accounted for in data cleaning, as described below.

Blood samples were collected every 2 h during the 40-h constant routine via indwelling intravenous cannula in the forearm or antecubital vein. Collections commenced 2 h post-wake and terminated 2 h before constant routine completion (19 samples per participant). Whole blood was collected and aliquoted into K2 EDTA tubes. Samples were stored and centrifuged (1300 ×  g, 10 min) at 4 °C within 30 min of collection. Plasma was extracted in 500 µL aliquots, stored on dry ice, and transferred to permanent storage at – 80 °C within 4–12 h. Of 437 possible sample collections across 23 participants, 416 were successfully collected and analyzed for metabolites. Number of samples collected per participant ranged from 14 to 19, with mean [± SD] = 18.1 ± 1.6 samples. Polar metabolite species were extracted using 1:1 acetonitrile/methanol and separated by HILIC high-performance liquid (SeQuant ZIC-pHILIC column + Agilent 1260), prior to detection by electrospray ionization mass spectrometry using an Agilent 6545 Q-ToF instrument. A total of 929 metabolite species were detected. A subset of the metabolomic data were published previously^13^.

### Metabolite analysis

Metabolite analysis followed a similar procedure to prior work.^13,40^ Plasma samples were thawed on ice; 20 μL aliquots were extracted using a 180 μL acetonitrile/methanol (1:1 v/v) solution containing 2 μM 13C-sorbitol, 2 μM 13C15N-AMP, and 2 μM 13C15N-UMP as internal standards. Samples were vortexed for 30 s, sonicated for 5 min at 4 °C, and then incubated for 10 min at 4 °C (using an Eppendorf Thermomixer). The samples were then centrifuged at 4,500 × g for 10 min at 4 °C, and 100 μL of the supernatant was transferred into a glass vial. Sample extracts (7 μL) were resolved on a ZIC®-pHILIC column (5 μm particle size, 150 × 4.6 mm, Merck SeQuant®) connected to an Agilent 1200 (Santa Clara, CA, USA) HPLC system running a 33 min gradient with mobile phases 20 mM ammonium carbonate (pH 9.0; Sigma-Aldrich; Solvent A) and 100% acetonitrile (solvent B) at a constant flow rate of 300 μL/min. Metabolites were detected by electrospray ionization using the Agilent 6545 Q-ToF MS system (Santa Clara, CA, USA) in negative all-ion fragmentor mode, which included three collision energies (0, 10, 20 V). The instrument was cleaned and calibrated weekly to ensure a mass accuracy of ± 2–5 ppm. The LC–MS processing was completed in two batches, with samples randomized by participant and time. A pooled biological quality control (PBQC) was run every eighth sample to monitor instrument performance. The PBQCs contained a 10 μL aliquot of each sample extract. To monitor background, solvent blanks were analyzed every 12 h.

Metabolite discrimination was based on accurate mass, retention time, and MS/MS fragmentation patterns of metabolites in standard mixtures. Area under the metabolite peak was used to obtain relative metabolite abundances, using MassHunter Quantitative Analysis B 0.7.00 (Agilent). Untargeted metabolite features were generated by XCMS centWave algorithm^41^ and refined in CAMERA to group related features by annotation of isotope and adduct peaks.

A total of 929 metabolite species were detected. A total of 416 out of a possible 437 plasma samples were collected and analyzed in 23 participants. A total of 303 out of a possible 323 plasma samples were collected and analyzed in 17 participants with core body temperature data.

For each individual sample, raw area count data were normalized relative to the median concentration of all metabolites across all samples. Metabolites with > 20% zero values within an individual were removed. Remaining data were then imputed for left-censored missing values using QRLIC (ImputeLCMD R package). Datapoints beyond ± 3 standard deviations were excluded (3,682 out of 386,464 metabolite datapoints, 0.95%). Metabolite time series were linearly detrended in each participant, by fitting a linear trend and subtracting fitted slope*time from each metabolite datapoint. This method was applied to control for wake-dependent or time-dependent increase or decrease in metabolite concentration^13^.

### Metabolite rhythmicity

Thirty-six-hour metabolite time series were assessed for 24 h rhythmicity by fitting the following (equation 1):

$$y = y_{0} + \alpha \sin \left( {\frac{2\pi }{\tau }t + \theta } \right),$$

where $t$ represents time since start of constant routine in hours, $y$ represents scaled metabolite concentration, fitted parameters are mesor ($y_{0}$), amplitude ($\alpha$), and phase ($\theta$), with fixed period of $\tau$ = 24 h. Parameters were fit with least-squares, using the ‘nlsLM’ package in R. Initial conditions were $y_{0} = 0$, $\theta = \pi$, and $\alpha$ equal to the standard deviation of the metabolite time series. Significant rhythmicity was defined by fits where the amplitude parameter ($\alpha$) was significantly different from zero at p < 0.05. This method was applied to each of 929 metabolite signals across 23 participants. We note that due to the relatively short metabolite time series (36 h), assessing significant fits with fixed $\tau$ = 24 h was expected to capture rhythmic metabolites with periods slightly above or below 24 h.

For assessment of variability in period, this fitting method was repeated with period ($\tau$) as a free-fitted parameter. The initial condition for metabolite period was set to $\tau$ = 24 h. Significant fits with periods in the range 16–32 h were included in the analysis of variability.

For both fixed and free-fitted period methods, each metabolite was defined as a circadian metabolite if it was significantly rhythmic in > 65% of participants in which metabolites were collected (i.e., ≥ 15 of the 23 participants). Sensitivity analyses confirmed findings were robust to this definition of circadian rhythmicity.

### Core body temperature data cleaning

Core body temperature records were cleaned by removing all epochs outside the approximate physiological range of human core body temperature at rest (< 36 °C or > 38 °C). The first 4 h after pill administration were excluded to minimize the impact of hourly liquid and food provision on the pill in the stomach. There was no overlap between core body temperature data in participants with two pills after applying this exclusion. The first hour after awakening was also excluded, allowing core body temperature to stabilize at waking levels. Remaining data were manually cleaned by removing epochs with sudden fluctuations (≥ 0.3 ºC increase or decrease across intervals of ≤ 0.5 h).

Six participants were excluded due to (a) regular reductions of logged pill temperature possibly resulting from hourly fluid and food intake (N = 4), (b) < 24 h recording length due to non-synchronization of pill and SEM device (N = 1), and (c) possible masking, observed as two apparent core body temperature minima separated by 16 h, coupled with outlying low weight (43.5 kg), low BMI (19.1), and complaints of feeling cold despite maintaining room temperature at 22 °C (N = 1).

Across 17 participants, cleaned data comprised 28.9 ± 3.7 h (M ± SD) of core body temperature epochs, across a collection interval of 35.6 ± 2.9 h, and 5.3 ± 3.1 h of core body temperature epochs were removed by manual cleaning. 5 of 17 participants had 4 h of data removed post-wake, and 12 of 17 participants had 1 h of data removed post-wake.

### Core body temperature fit and amplitude extraction

Core body temperature time series were fit using a two-harmonic equation (equation 2):

$$y = y_{0} + \alpha_{1} \sin \left( {\frac{2\pi }{\tau }t + \theta_{1} } \right) + \alpha_{2} \sin \left( {\frac{4\pi }{\tau }t + \theta_{2} } \right) ,$$

where $t$ represents time since start of constant routine (h), $y$ represents scaled metabolite concentration, fitted parameters are mesor ($y_{0}$), amplitudes of the two harmonics ($\alpha_{1}$ and $\alpha_{2}$), and phases of the two harmonics ($\theta_{1}$ and $\theta_{2}$), with $\tau$ = 24 h. Previous analyses suggest this two-harmonic Fourier approximation is sufficient to describe core body temperature rhythms under constant routine conditions^42^. Minimum and maximum of core body temperature rhythms were defined as the fitted minimum and maximum of the two-harmonic equation. Core body temperature amplitude was defined as half of the difference between the fitted minimum and maximum of the core body temperature rhythm across 24 h (i.e., composite amplitude).

### Statistical analyses

Simple linear regression models were applied to test the relationships of the number of rhythmic metabolites and variability of free-fitted metabolite periods with core body temperature amplitude (two-tailed, α = 0.05, N = 17 for both models).

## Data Availability

The data that support the results of this study, including core body temperature time series and metabolite area-count time series, will be made available by the corresponding author upon reasonable request.
